# Modeling Rett Syndrome with Human Pluripotent Stem Cells: Mechanistic Outcomes and Future Clinical Perspectives

**DOI:** 10.3390/ijms22073751

**Published:** 2021-04-03

**Authors:** Ana Rita Gomes, Tiago G. Fernandes, Joaquim M.S. Cabral, Maria Margarida Diogo

**Affiliations:** 1 Department of Bioengineering and IBB-Institute for Bioengineering and Biosciences, Instituto Superior Técnico, Universidade de Lisboa, Av. Rovisco Pais, 1049-001 Lisboa, Portugal; ana.b.gomes@tecnico.ulisboa.pt (A.R.G.); tfernandes@tecnico.ulisboa.pt (T.G.F.); joaquim.cabral@tecnico.ulisboa.pt (J.M.S.C.); 2 Associate Laboratory i4HB-Institute for Health and Bioeconomy, Instituto Superior Técnico, Universidade de Lisboa, Av. Rovisco Pais, 1049-001 Lisboa, Portugal; 3 Instituto de Medicina Molecular-João Lobo Antunes, Faculdade de Medicina da Universidade de Lisboa, 1649-028 Lisboa, Portugal

**Keywords:** Rett syndrome, MeCP2, neurodevelopmental disorders, hPSCs, hiPSCs, 2D models, organoids

## Abstract

Rett syndrome (RTT) is a neurodevelopmental disorder caused by mutations in the gene encoding the methyl-CpG-binding protein 2 (MeCP2). Among many different roles, MeCP2 has a high phenotypic impact during the different stages of brain development. Thus, it is essential to intensively investigate the function of MeCP2, and its regulated targets, to better understand the mechanisms of the disease and inspire the development of possible therapeutic strategies. Several animal models have greatly contributed to these studies, but more recently human pluripotent stem cells (hPSCs) have been providing a promising alternative for the study of RTT. The rapid evolution in the field of hPSC culture allowed first the development of 2D-based neuronal differentiation protocols, and more recently the generation of 3D human brain organoid models, a more complex approach that better recapitulates human neurodevelopment in vitro. Modeling RTT using these culture platforms, either with patient-specific human induced pluripotent stem cells (hiPSCs) or genetically-modified hPSCs, has certainly contributed to a better understanding of the onset of RTT and the disease phenotype, ultimately allowing the development of high throughput drugs screening tests for potential clinical translation. In this review, we first provide a brief summary of the main neurological features of RTT and the impact of MeCP2 mutations in the neuropathophysiology of this disease. Then, we provide a thorough revision of the more recent advances and future prospects of RTT modeling with human neural cells derived from hPSCs, obtained using both 2D and organoids culture systems, and its contribution for the current and future clinical trials for RTT.

## 1. Introduction

Rett syndrome (RTT) is a neurodevelopmental disorder with higher incidence in females, causing, in the majority of cases, mental retardation. The incidence ranges from 1:10,000–20,000 live births, with sporadic cases (9%) found in males [1]. The clinical features start to be noticeable after 6 to 18 months of normal development after birth [2]. The developmental regression includes several neuropathological features that compromise the brain function, language and learning, motor disabilities, as well as repetitive stereotyped hand movements, seizures and autistic behavior.

**Figure 1:**
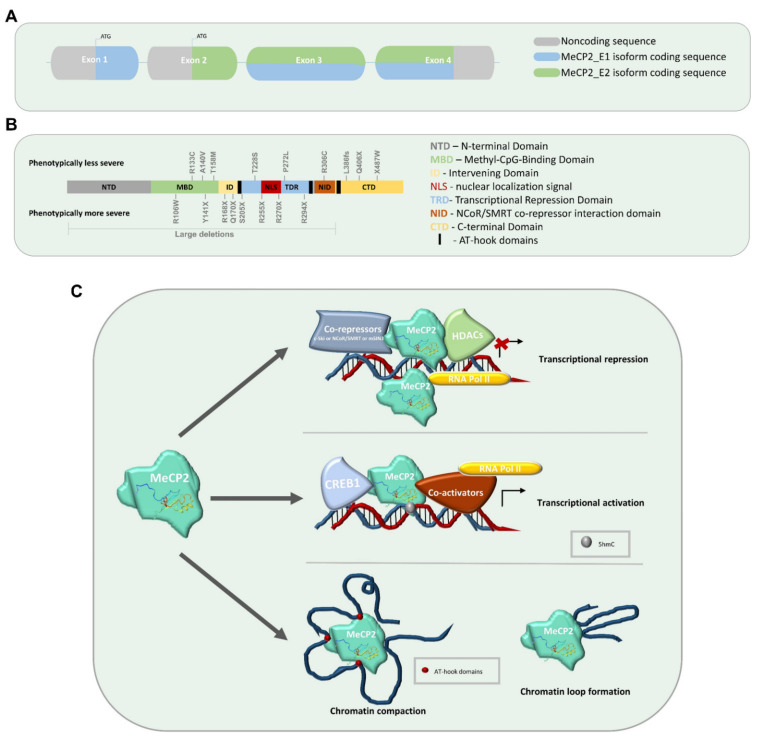
(**A**) Schematic view of the MeCP2 gene structure. The figure shows a representative structure of the four exons that constitute the MeCP2 gene, and the respective isoform coding sequence for each MeCP2 proteins isoforms, MeCP2_E1 and MeCP2_E2. (**B**) Schematic representation of MeCP2 protein domains. Each domain is annotated with the more common and frequent mutations found in the public database http://mecp2.chw.edu.au (accessed at 10 March 2021). On the upper part are annotated the mutations associated with milder phenotypes, while in the bottom are mutations associated with the more severe phenotypes of RTT. (**C**) Schematic view of the major roles of MeCP2. MeCP2 is capable of repressing or activating the transcription of several target genes, recruiting co-repressors and co-activators. Some known co-repressors are c-Ski, N-CoR/SMRT and mSIN3A. MeCP2 also represses the rate of RNA polymerase (Pol II) transcription initiation. One known mechanism of MeCP2 gene activation is through direct binding to CREB1. MeCP2 also induces transcriptional modifications by altering chromatin conformation and by promoting the formation of the chromatin loop.

The causative gene has been identified in 90% of the cases as the one encoding for the methyl-CpG binding protein 2 (MeCP2), located in the X chromosome [3]. The MeCP2 gene has 4 exons, which are alternatively spliced and produce two transcripts, MeCP2-E1 and MeCP2-E2 [4], as represented in Figure 1A. The molecular mechanism behind the impact of each MeCP2 mutation during brain development and function still remains elusive. Nevertheless, it is known that the severity of the disease may be influenced by the type and location of the each MeCP2 mutations. Around 90% of the patients are diagnosed with a point mutation or small deletions in the MeCP2 gene (http://mecp2.chw.edu.au/, http://www.biobank.unisi.it, http://www.MeCP2.org.uk/ (accessed at 10 March 2021)), causing the symptomatic heterogeneity observed in classical RTT patients. For example, early-truncating mutations (such as R168X, R270X, R255X), occurring in the methyl-CpG-binding domain (MBD) and in the transcriptional repression domain (TRD), could eventually be responsible for the complete loss of MeCP2 function, which is associated with a more severe phenotype [5] (Figure 1B). On the other hand, late-truncating mutations in the C-terminal domain or in missense mutations (R133C, R294X, R306C, T158M) may be less severe, as MeCP2 function is not completely abolished. Several studies have reported genotype–phenotype correlations [6,7,8], which are essential findings to understand the several mutation-specific MeCP2 molecular mechanisms. This is crucial knowledge, as the design of a proper clinical trial and of an appropriate therapeutic intervention must have in consideration the phenotypic variability associated with the different mutations.

Despite the phenotypic variability caused by MeCP2 mutations, random X-chromosome inactivation (XCI) is still the major cause of distinct phenotypic severities in RTT. In a large number of cases, the resulting somatic mosaicism is defined by an equal number of cells with active maternal or paternal X-chromosome, which results in normal and mutant MeCP2 randomly distributed, for example within the brain [9]. However, the X-chromosome inactivation is a very complex phenomenon, difficult to understand, since it was also reported to occur skewed or randomly, and the mutant allele may have a paternal or a maternal origin. Some studies which analyzed the inactivation pattern indicate that the severity is often dependent on the percentage of inactivation verified on the paternally derived X chromosome [10]. Other studies examined the XCI patterns in the regions that are predominantly affected by RTT. By using samples from cortical brain tissue collected from RTT patient autopsies, it was found that in some cases XCI was balanced in cortical tissues, whereas in others XCI was randomly dispersed throughout the cortex [11] and in the cerebellum [12]. Overall, the data are not conclusive in what concerns the XCI-related severity, and other factors such as epigenetic-related mechanisms and mechanisms associated with MeCP2-target genes may also contribute to the phenotypic heterogeneity among different patients [8].

Considerable progress in understanding the mechanisms of RTT has been made in recent years by conducting studies in animal models, the male homozygous mice (MeCP2_/y) model being the most frequently used due to the early development of severe phenotype and the inexistence of a mosaic pattern [13]. However, the clinical relevance of the male mice model may be low due to the fact that it does not represent the features of the female patients, the ones predominantly affected. During the past decade, the motivation to use alternative models, which more accurately resemble the severity and the features of RTT, has been growing. In this context, human in vitro cellular models, both cultured in the format of 2D monolayer or as 3D aggregates, have been developed by using human-induced pluripotent stem cells (hiPSCs) reprogrammed from RTT patient somatic cells as the initial cell source.

In this review we will briefly summarize the several neurological characteristics of RTT and the role of MeCP2 in RTT pathophysiology. As the main focus, we will cover the most recent breakthroughs of RTT modeling using human in vitro cellular models obtained by differentiating hPSCs, both hiPSCs or human embryonic stem cells (hESCs), and their use for studying the molecular features of the disease, as well as their contribution to the design of ongoing or future clinical trials.

## 2. Rett Syndrome—The Pathophysiology

RTT was described for the first time by Dr. Andreas Rett, in 1966, upon observation of two girls with stereotypic hand movements, impaired neural development and brain atrophy [14]. Later, in 1999, Huda Zoghbi’s laboratory found that disease-causing mutations in MeCP2 were the main cause of RTT in patients, accounting for around 90% of the cases, with the majority of them occurring due to de novo mutations [15]. The RTT-like phenotype, associated with mutations in CDKL5 [16] and FOXG1 [17] genes, occurs in 10% of the disease cases.

After a normal developmental stage, until 6–18 months of life, a subsequent regression is observed in RTT patients, and a delay in development is the first phase of disease symptomatology [2]. After a period of development stagnation, a rapid regression is observed in the subsequent 4/5 years, which is characterized by the onset of additional symptoms, such as ataxia, associated with a strong motor disability, and mental retardation. A stationary/plateau phase follows that serious regression period, and with therapeutic intervention, some patients are able to regain some abilities [18,19]. However, depending on the severity, it is also possible that in the last phase of the disease some patients develop respiratory difficulties, abnormal sleeping, scoliosis [20], cardiac dysrhythmias and, over time, motor deterioration and neurodegenerative associated problems, like Parkinson-related dysfunction [21]. Due to the severity of the symptoms, the life span of patients may be decreased, with sudden deaths being reported [22]. However, with proper clinical care, around 70% of patients are able to reach the age of 50 [22]. Still, the central nervous system has been reported to be the main affected area of the patient’s body, first detected by the deceleration of head growth in the majority of patients [23]. Likewise, previous studies reported a reduced volume of the cerebellum and cerebral cortex of RTT patients [24].

As noted above, the clinical severity of the RTT phenotype can dramatically vary between patients. For example, the incidence of seizures, which usually occurs around 4 years of age, is present in around 80% of the cases, being reported more frequently in patients with early-onset and more severe phenotype of RTT [25,26]. For example, missense mutations are associated with early onset and severe epileptic phenotype [27]. In association with RTT mutations, it was observed the influence of brain-derived neurotrophic factor (BDNF) polymorphism, which is mediated by MeCP2 (see the following sections for a more complete review), in increasing the risk and severity of seizures [28]. Moreover, early onset of epilepsy and daily occurrence were demonstrated to represent a higher risk for suffering from drug resistance, whereas 20–40% of patients become refractory to treatment [29,30].

This rare monogenic disease is also one of the most common causes for intellectual disability in females. However, male patients have also been identified with RTT [1]. Sporadic cases of RTT in males, mostly caused by MeCP2 genetic point mutations (with a few cases of FOXG1 or CDKL5 mutations also being reported), have been observed to exhibit neonatal encephalopathy and severe intellectual impairment, with death occurring in infancy [31,32,33]. Similarly to females, the mutation type and its location in the gene exerts a significant influence in determining the disease severity [33]. In addition to MeCP2 mutations, abnormal karyotypes have also been associated with RTT male cases, such as the Klinefelter syndrome (47, deXXY) [34] or even somatic mosaicism [32,35]. The cases of non-fatal occurrence in RTT males, or the mild features observed within the male MeCP2 mutations, could be explained by several mechanisms. One hypothesis is the occurrence of somatic mosaicism without any karyotype alteration [32]. Other possible explanations may rely on the occurrence of de novo spot mutations compatible with adult life, which have been never observed in RTT females [36], or the occurrence of MeCP2 gene duplication [37]. MeCP2 duplication is also considered a clinical neurodevelopmental syndrome, resulting in a genotype–phenotype correlation, causing severe features such as mental retardation [38,39]. MeCP2 duplication is more frequently detected in males than in females, being the female dosage alleviated by the phenomenon of XCI. Therefore, clarifying the consequences of different MeCP2 levels in the manifestation of the disease must be a step forward into the clinical treatment of RTT.

The two utmost MeCP2 alterations, MeCP2-knockout (KO) and MeCP2 duplication, have been extensively modelled in both mouse models [40] and cellular models [41]. Such studies have displayed a huge amount of information concerning the role of MeCP2 in several neurodevelopmental mechanisms and consequently the associated neurological features of the disease. However, the development of models capable of mimicking all the aforementioned aspects of RTT, particularly XCI inactivation, MeCP2 levels, mutation-type, and others, in a patient-specific manner, have greatly increased in interest. These humanized models improve the understanding of MeCP2-related disease mechanisms and ultimately contribute to the development of novel therapeutic approaches for RTT.

## 3. An Overview of MECP2 Role as a Transcriptional Regulator

MeCP2 has been proved essential during brain development and function, influencing neuronal differentiation, maturation and synaptic plasticity. The wide symptomatic variability observed in RTT may also be affected by variations in the expression of other genes that are regulated by MeCP2. Depending on the circumstances, MeCP2 can act as an activator or as an inhibitor of gene expression. Several studies have been focusing on the mechanisms behind the role of MeCP2 as a transcriptional regulator, and the most recent findings have been extensively reviewed elsewhere [42,43,44,45,46,47].

The transcriptional modulator role of MeCP2 is complex since it shows distinct binding sites and patterns depending on the neurodevelopmental and adulthood stage. MeCP2 mutations and time-variations observed in epigenetic regulation of gene expression may contribute to the delayed onset of RTT symptoms and consequently to the phenotypic severity and variability observed.

MeCP2 is composed of multiple domains, including the N-terminal domain (NTD), the methyl-CpG-binding domain (MBD), the intervening domain (ID), the transcriptional repression domain (TRD), the nuclear co-repressor (NCOR)-silencing mediator of retinoic acid and thyroid hormone receptor (SMRT) interacting domain (NID), located within the TRD, and the C-terminal domain (CTD) [48,49] (Figure 1B).

The most relevant domains are the MBD and TRD, since they are associated with DNA binding and transcriptional repression, being the most frequent mutations associated to the disease located within these domains. MeCP2-mediated transcriptional repression is due to the interaction of MeCP2 with methylated DNA, mainly at CG and CA dinucleotides [50]. Studies have shown that MeCP2 directly binds co-repressors such as c-Ski, N-CoR/SMRT and mSIN3A [49,50,51,52]. The histone deacetylases (HDAC) are recruited to the methyl-CpG regions to promote the suppression of transcription (Figure 1C). Several studies have been focusing on the mechanism of MeCP2 regulation of transcriptional repression. Recent experiments showed that MeCP2 represses nascent RNA transcription, by interacting with the NCoR co-repressor complex [53]. MeCP2 was observed to act in the starting sites of transcription, limiting the synthesis of mRNAs by repressing the rate of RNA polymerase (Pol II) transcription initiation, mostly for the highly methylated long genes present in the brain (Figure 1C). Additionally, MeCP2 levels are significantly increased in the adult brain, being also observed to have high affinity for binding to methylated cytosine (mC) at mCH dinucleotides (H = A, C, or T). The maturation and activity of neurons are regulated by the non-CG DNA methylation pattern [54,55]. Thus, MeCP2 strongly contributes for the transcriptional regulation (e.g., BDNF) in the adult brain [50,54]. Chromatin immunoprecipitation-sequencing analysis (ChIP-seq) of MeCP2 demonstrated its higher affinity for genes with increased density of mCA levels, and simultaneously, MeCP2-KO neurons showed an upregulation of mCA enriched long genes [56]. Recently, it was established that mCA patterns in neural cells are associated with chromatin folding regions and MeCP2 often tends to bind to topologically associating domains (TADs) present in those mCA sites [57]. The enhancer activity in the previous domains is repressed and the target gene expression is deregulated.

An important research performed by Chahrour et al. identified MeCP2 not only as a modulator of the transcriptional repression, but also as a transcriptional activator [58]. The authors demonstrate this activation pattern by mass spectrometry analysis, showing that MeCP2 directly activates the cAMP response element-binding protein 1 (CREB1) (Figure 1C), a major transcriptional activator [58], by associating with CREB1 at the promoter of an activated target, such as BDNF, SST, OPRK1, GAMT, GPRIN1 [58].

MeCP2 levels are also essential for the regulation of the 3D structure of neuronal chromatin [59] (Figure 1C), since alterations within heterochromatin structure displayed different phenotypic severities of RTT [60]. Moreover, a few studies have proved that impaired MeCP2 was responsible for triggering stem cell senescence, which could be implicated in an aberrant progenitor’s self-renewal, consequently exhausting, for example, the neural stem cell pool or the capacity for bone remodeling [61,62].

As briefly reviewed in this section, MeCP2 presents a variety of different functions and acts by several distinct mechanisms, which are dependent on the molecular context, as shown in Figure 1C. Thus, the complexity found in RTT is not surprising. Nevertheless, the majority of the studies described here were performed using null MeCP2 mouse models, missing the recapitulation of mosaicism and the pattern associated to each MeCP2-mutation. Despite the importance of such models for RTT research, ongoing and further studies using advanced in vitro humanized brain models of RTT will certainly improve the understanding of RTT mechanisms and RTT patient-specificity, in what concerns phenotype variability and disease severity.

## 4. Modelling RTT with hiPSCs

As previously mentioned, during the past few decades, RTT has been studied in transgenic animal models, such as mouse models. Mouse models are able to recapitulate some features of human RTT, being in the last decade the major source of new findings regarding RTT mechanisms and pathways. However, the translatability toward humans, due to the milder phenotype observed in female patients, and the later onset of the disease in rodent models is not clear [63]. Intact human brains/pos-mortem samples have also been studied, providing important clues related to the gene expression profile associated to the disease pathology [64]. Besides the difficulty in accessing these samples, they generally represent end-points of RTT and they cannot be genetically manipulated or be used as models for studying disease-associated mechanisms and functional studies, or even for high-throughput drug screening. To overcome these limitations, the derivation of human embryonic stem cells (hESCs) from blastocysts and the reprograming of human somatic cells into hiPSCs have provided the most promising cell sources for generating in vitro culture platforms for modeling RTT.

RTT patient-specific hiPSC lines, together with gene edited hiPSCs or hESCs, have been intensively used to investigate the consequences and the phenotypic relevance of the localization of each specific mutation across the different MeCP2 domains [65]. Furthermore, by efficiently correcting mutated MeCP2 using genome editing tools, such as CRISPR/Cas9, it may be possible in the future to develop gene therapy strategies for RTT [66]. Prior to phenotypic studies of RTT with hiPSCs-derived models, fundamental research has been performed to understand the X-inactivation patterns upon hiPSC reprograming and during differentiation, which generate hiPSC-derived cultures with epigenetic heterogeneity [67,68]. This knowledge contributed to a better understanding of the different patterns and the mosaicism phenomenon in patients and, importantly, it is currently used for obtaining isogenic control hiPSC lines without any genomic manipulation [69].

RTT is a “multi-systemic” pathology, but the loss of MeCP2 specially impacts different stages of brain development and function. Thus, neurological features associated with RTT are the best documented and the ones raising more interest. It has been demonstrated that RTT genetically modified hPSCs and RTT patient-specific hiPSCs, carrying specific mutations and patient’s specific genetic backgrounds, are able to provide realistic in vitro recapitulation of the neurodevelopmental process. Several methodologies have been established to obtain 2D cultures of neural cells or, more recently, 3D cell aggregates mimicking a brain-like structure (brain organoids) from hiPSCs. In the following sections we review the several in vitro models that have been used for studying RTT (2D and 3D formats), and the most encouraging outcomes obtained from using these model systems.

### 4.1. 2D-Based Neuronal Differentiation Models

In the last decade, several groups have intensively modelled and studied RTT using 2D systems of differentiating hPSCs that recapitulate several aspects of both molecular and functional RTT phenotypes. Moreover, hPSCs-derived 2D models have been used for understanding the role of MeCP2 in human neurons, by directly altering neural function, or by indirectly controlling other related-gene targets. Technological advances have improved the amount and quality of the generated data, and more recently, by using high-resolution quantitative analyses, it has been possible to study RTT alterations in terms of global biological processes and the molecular mechanisms involved in the onset of the disease. Finally, a great portion of these studies aimed to discover novel potential candidate drugs through drug screening assays.

#### 4.1.1. Evaluating Altered RTT Phenotype

Marchetto and colleagues were the first to generate a RTT hiPSCs-based model including four distinct MeCP2-associated mutations [70]. They started by producing embryoid bodies (EBs), from which neural rosettes were manually collected, dissociated and re-plated, and then induced them to generate neural progenitors and undergo maturation into neurons. Moreover, the authors observed that RTT-derived neurons exhibited impaired maturation, including the presence of fewer synapses, smaller soma size, altered calcium signaling, functional defects in firing activity and excitatory/inhibitory (E/I) imbalance [70]. In a similar culture approach, neurons derived from a R294X-hiPSCs mutated cell line were found to display a smaller nuclear size in RTT neurons [71]. Further studies from other authors using neurons differentiated from mutant and biallelic RTT hiPSC lines demonstrated lower levels of TUJ^+^ neurons and lower levels of sodium (Na^+^) channels, when compared with control neuronal cultures [72].

The first study correlating MeCP2 mutations and the two protein isoforms, MeCP2_E1 and MeCP2_E2, was performed by Djuric and coworkers [73]. The study was conducted using exon-deletion-isoform RTT hiPSC lines, and the respective isogenic control, upon 2D differentiation into cortical neurons. The authors observed that mutant neurons exhibited a decrease in soma size, reduced dendritic complexity and decreased cell capacitance. Interestingly, the phenotype was only rescued by transgene-induced expression of MeCP2_E1, but not of MeCP2_E2. Moreover, the observed impaired maturation was due to defects in action potential generation, exhibiting a smaller amplitude and a longer time course, which could be molecularly related with a decrease in voltage-gated Na^+^ currents [73].

After these initial studies of RTT modelling using hiPSC lines, the rapid progress in understanding the processes of neural lineage specification allowed the development of more standardized, chemically defined, robust and reproducible methodologies for neural differentiation of hPSCs. Consequently, dual-SMAD inhibition was used for neuroectodermal specification of hiPSCs and hiPSC-based RTT modeling [74]. Fernandes et al. derived neurons from a female (R306X) RTT patient-specific hiPSC line, under 2D chemically defined conditions, using the dual-SMAD inhibition protocol and vitronectin as the adhesion matrix. By employing this methodology, it was possible to generate TUJ1^+^ and MAP2^+^ neurons, which revealed alterations in the number of neuronal projections obtained in RTT neurons [75].

#### 4.1.2. Understanding MeCP2′s Molecular Functions

The search for potential MeCP2 molecular targets has also been pursued by modelling RTT using hESCs and RTT hiPSC lines. For example, neurons derived from patient-specific RTT hiPSC lines, carrying the R306C and 1155D32 mutations, were supplemented with Choline, a precursor of membrane components [76]. Accordingly, Choline altered the lipid profile of the membrane of RTT neurons, and consequently rescued neuronal soma size and synaptic input, by increasing the frequency of spontaneous excitatory postsynaptic currents [76].

In another example of this type of studies, gene editing was used for the derivation of MeCP2-KO hiPSCs or lines carrying the T158M and V247fs mutations. Upon differentiation into forebrain neurons, these cells exhibited phenotypic alterations including a reduced neuronal growth, a reduced dendritic complexity, and increased fragmentation and reduced mitochondrial membrane potential [77]. Together, these observations were correlated with reduced CREB levels, a transcription factor known to indirectly regulate MeCP2 levels, and from which overexpression rescues the phenotype associated with RTT mutant neurons. Moreover, the authors used a translational approach, by which the neuronal phenotype was rescued by increasing the phosphorylation of CREB using the compound Rolipram [77].

Another role of MeCP2 as a molecular regulator was observed to occur with the cell adhesion molecule L1. In fact, reduced expression of L1 was observed in RTT hiPSCs-derived neural precursor cells, and correlated with decreased neuritogenesis [78].

The neuron-specific K^+^-Cl^−^ cotransporter 2 (KCC2) is a critical downstream gene target of MeCP2, by inhibiting the RE1-silencing transcriptional factor (REST) [79]. Upon neuronal differentiation of male RTT hiPSCs lines presenting the Q83X mutation, these cells revealed deficits in KCC2 expression, linked with impaired GABA functional switch from excitation to inhibition. This work suggested a possible therapeutic strategy for RTT based on the KCC2 overexpression or insulin-like growth factor-1 (IGF-1) treatment, which is known to interfere with a GABA functional switch [79]. Recently, a high-throughput screening (HTS) approach using MeCP2-null RTT hESCs screened several small molecules capable to increase KCC2 expression [80]. The most promising candidates were KW-2449, an inhibitor of fms-like tyrosine kinase 3 (FLT3), and BIO, an inhibitor of the glycogen synthase kinase 3 β (GSK3β) pathway. In fact, these small-molecules efficiently rescued the GABA functional switch, excitatory synapses and E/I balance by increasing the proper levels of KCC2, which regulates intracellular [Cl^−^] in neurons [80].

As previously described, the over dosage of MeCP2 protein can also trigger RTT-like symptoms. Interestingly, Nageshappa et al. studied hiPSCs-derived cortical neurons, obtained from a patient with MECP2-duplication disorder, having observed an increased synaptogenesis and dendritic arborization complexity, associated with increased levels of the dendritic branching modulator CUX1, and also an altered network synchronization. Moreover, by testing a library of compounds acting in epigenetic pathways, the authors validated a potential clinical candidate for RTT treatment, the HCAD inhibitor NCH-51 [41].

#### 4.1.3. High-Content Molecular Analysis of RTT Cells

High-content molecular analysis has also been used to define the molecular pathways involved in RTT. Among these, transcriptomic and proteomic analysis provided valuable insights regarding neurodevelopment, and have been recently used to characterize the molecular signatures of RTT pathology. In this context, in one study, RTT neural progenitors and neuronal cells derived from hiPSCs were used for bulk transcriptomic analysis. This revealed a direct correlation between P53 induction and the dendritic branching defects observed in RTT. Moreover, P53 targeted genes such as P21, GADD45, DDIT4, and DDB2, were also observed to be upregulated in RTT neurons. Additionally, other cell-stress pathways were observed to be upregulated in MECP2 null interneurons, particularly genes associated with the senescence secretory program [81].

Another study accessed the transcriptomic profile of two different mutated hiPSCs cell lines, R306C and T158M, contributing to add additional molecular insights [82]. Interestingly, these results indicated impairments in the circuit of GABAergic neurons, more specifically correlated with an atypical reduction in acetylated α-tubulin, as a consequence of Histone Deacetylase 6 (HDAC6) overexpression. Moreover, treatment with ACY-1215, a HDAC6 inhibitor, was able to rescue the abnormal levels of HDAC6 in RTT cells.

In order to assess the impact of MeCP2 mutations specifically in hiPSC-derived interneurons (INs), a recent study employed a protocol for generating medial ganglionic eminence (MGE) and functional INs [83]. It was possible to observe several phenotypic differences between INs generated from MeCP2-R133C, a male hESC line carrying the mutation R133C (C397T) induced by CRISPR-Cas9, and the respective isogenic control. Particularly, INs from the RTT line displayed a decrease in neurite growth and a decreased soma size. These cells also showed defects in electrophysiological properties, namely the absence of discharging APs, a low degree of synchronization, and a lower amplitude in spiking activity. At day 74 of differentiation, results from RNA sequencing of mature RTT INs revealed an extensive dysregulation in gene expression, mainly related with biological processes associated with axon/dendrite growth, synapse development and ion channel activities that are calcium-related. The use of JQ1 (a thienotriazolodiazepine that interferes with the epigenetic pathways, and whose primary target is bromodomain 4 (BRD4)) revealed promising results in rescuing the altered phenotype. Particularly, an increase in the expression of genes involved in the regulation of neuronal structure and function, for example, IGF1, KCC2, and mGlu7, was observed. As a result, the mechanistic understanding of this response was revealed, since MeCP2 and BRD4 co-regulate the transcription of common genes, and BRD4 binding levels were decreased following treatment with JQ1, resulting in the normalization of gene expression in the RTT mutant INs [83].

As another example, a spectrometry-based quantitative proteomic analysis was performed during neural differentiation of RTT hiPSCs, using the dual-SMAD inhibition protocol [84]. The data revealed interesting alterations in proteins involved in several signaling pathways, when compared with isogenic controls. These alterations were related with downregulation of proteins involved in synaptogenesis, dendritic morphology, excitatory postsynaptic potential, histone acetylation, nervous system development and forebrain development pathways, while upregulated proteins were shown to be involved in insulin receptor signaling, cell–cell adhesion, acyl-CoA metabolic process, actin cytoskeleton organization, apoptosis, DNA repair, oxidation and metabolism-related pathways. This proteomic analysis demonstrated strong evidence of molecular changes occurring early during neurodevelopment, before the onset of RTT disease.

Another pioneering study also investigated the proteomic profile of the exosomes present in the conditioned medium of MeCP2 loss-of-function neurons derived from hiPSCs [85]. Notably, neurodevelopmental signaling proteins, mainly associated with neuronal maturation, axonal guidance and synaptogenesis, were upregulated in the isogenic control-derived exosomes, when compared with the RTT ones. Interestingly, upon culture of RTT neurons with control exosomes, the authors observed increased puncta densities (Synapsin1 staining) and increasing synaptogenesis, whereas spike recordings revealed an improvement in neuronal activity with higher network synchronization.

**Figure 2:**
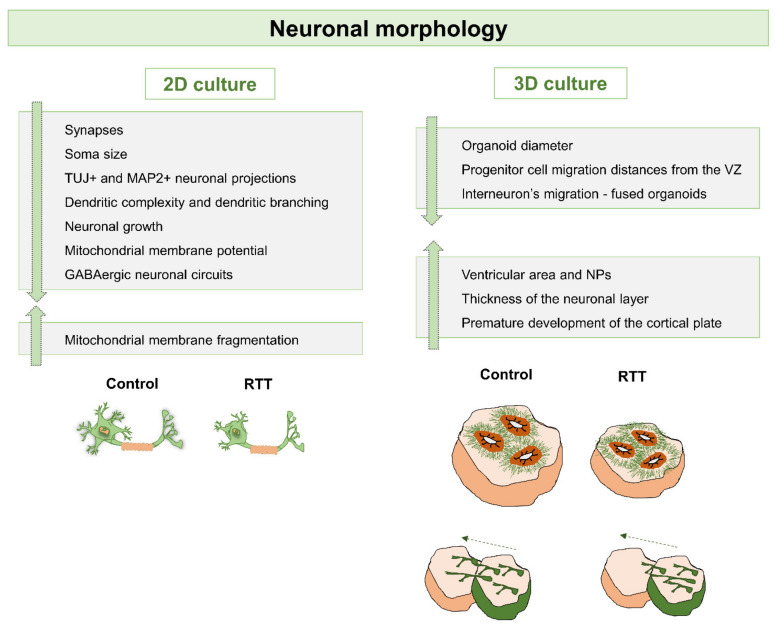
Schematic view of neuronal morphology-related alterations observed in 2D and 3D models of RTT derived from hiPSCs. Neuronal structures, such as synapses, dendrites, neuronal projections, mitochondria, and some cortical layers have been observed to be altered in RTT. Highlighted in this figure are the alterations in the cell migration process and organoid size, as well as smaller cell soma size, altered dendrites and smaller neuronal projections of neuronal cells.

**Figure 3:**
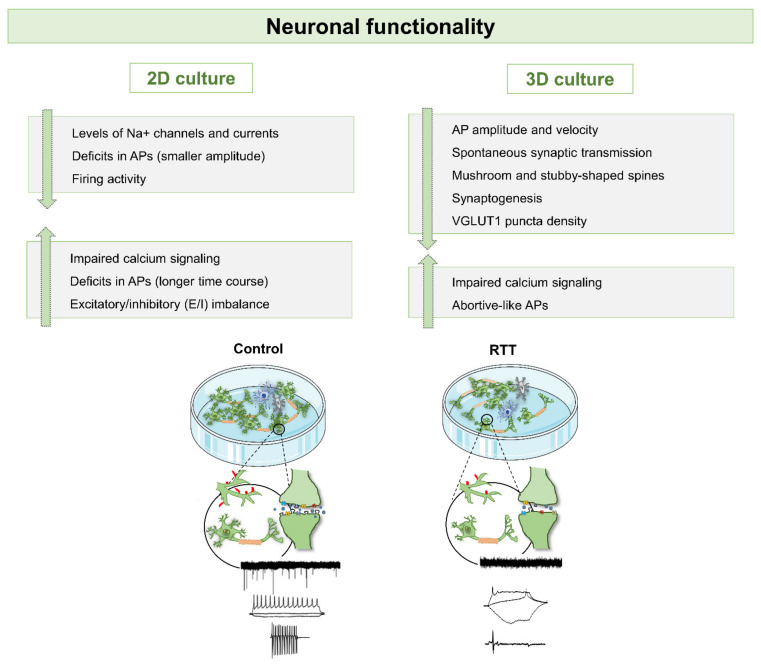
Schematic view of functional alterations observed in 2D and 3D cultures of RTT derived from hiPSCs. Similar functional alterations have been observed in 2D and 3D RTT models, mainly the deficits related with action potential (APs) generation and deficits in calcium signaling. Both synaptogenesis and spine shape alterations revealed the typical immature functionality observed in RTT cellular systems. Examples of functional neuronal deficits highlighted are the availability/functionality of ion channels and deficits in neurotransmitters and dendritic spines.

**Figure 4:**
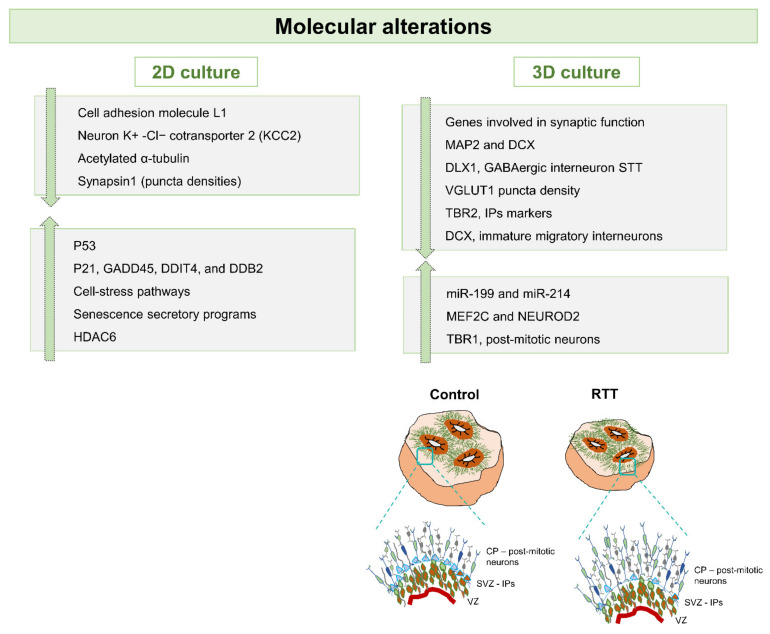
Schematic view of RTT-associated molecular alterations observed in both 2D and 3D cultures of brain cells derived from hiPSCs. Genes related with both GABAergic interneurons (DLX1) and glutamatergic cortical neurons (VGLUT, TBR1) have been observed to have altered expression in RTT models. Additionally, alterations in molecules associated with cell stress and apoptosis, and involved in synaptic function are also highlighted. The schematic view highlights the differences in cortical layer structures, mainly the increase in the thickness of the cortical plate (CP) and the absence/decrease of intermediate progenitors (IPs) in the subventricular zone (SVZ), laying adjacent to the ventricular zone (VZ).

Overall, these studies demonstrated that 2D based hiPSCs-derived neuronal models are a powerful tool to reveal the mechanisms associated with different MeCP2 mutations and consequently different protein levels [86]. Despite the fact that these models are still considered very simplistic, together they had a tremendous impact since they allowed the understanding of structural, molecular and physiological RTT-related phenotypes during early developmental stages of the disease (Figure 2, Figure 3 and Figure 4). Importantly, it was possible to observe aspects that are common to the different RTT mutations and genders, and also to access specific phenotypic characteristics of different RTT conditions.

### 4.2. 3D Brain Models

Modelling of RTT has also been recently achieved using 3D models of the human brain, firstly by using scaffold-based 3D models and more recently through the use of brain organoids. Recent advances combining stem cell biology and engineering made possible the generation of brain organoids. These 3D cellular structures, formed by self-assembling of hundreds of thousands to millions of cells, attempt to mimic the complex architecture and function of the brain. Brain organoids derived from hiPSCs have been making a huge impact in biomedicine by providing a better understanding of human brain development and circuit formation, and consequently the associated disorders [87]. In the initial approaches for generation of brain organoids, these 3D structures were generated from neurospheres [88] or embryoid bodies (EB) [89], without any major external interference, and were composed of a very heterogeneous variety of cell lineages. More recently, however, region-specific brain organoids have been generated using guided approaches relying on the use of specific small molecules and growth factors throughout the differentiation process [90,91,92]. In this section, we will review the recent breakthroughs in the study of RTT-associated phenotypes, and the respective mechanisms, using hiPSC-derived 3D brain models with a major emphasis on 3D brain organoids.

#### 4.2.1. Scaffold-Based 3D Models

A pioneering 3D culture system used for studying RTT consisted in a layered hydrogel, a cross-linkable methacrylate-modified hyaluronic acid [93]. This soft matrix was shown to recreate the 3D environment, increasing cell-to-cell interactions and promoting neuronal maturation, due to the material properties that mimic the extracellular matrix (ECM) composition. This scaffold allowed a more refined spatial control over cell location, and also the detailed observation at the single cell level, proving to be an advantageous system for the study of the neuronal migration process. By using NPCs derived from distinct male hiPSCs lines, specifically with the Q83X and the N126I mutations, it was possible to observe delayed migration of RTT astrocytes and neurons, when compared with control cells. These results indicate intrinsic impairments in RTT NPC migration, and consequently, after maturation, defects in neurite outgrowth and lower synapse punts were observed in RTT neurons.

Later on, a distinct 3D system was employed for studying the effect of electrical stimulation in neuronal maturation of RTT hiPSCs-derived NPCs [94]. The study used a conductive 3D graphene scaffold, recreating the 3D environment and simultaneously allowing electrical stimulation of RTT NPCs. When stimulated during early neuronal maturation, RTT neurons showed increased levels of TUJ1 and DCX and enriched soma size, indicating possible improvement in the RTT phenotype at the initial stage of neuronal maturation.

The aforementioned systems allowed the recreation of in vivo environmental conditions to a degree that could not be attained in standard 2D culture, mainly by promoting complex cell–cell interactions, or by mimicking physical stimuli particular to the brain. The use of 3D platforms thus creates more realistic spatial distribution and organization of neural networks in vitro. Therefore, these systems make possible the study of cell migration, the screening of chemical factors, and the evaluation of physical stimulus in 3D settings, and will result in responses that are closer to the ones observed in vivo, when compared with the 2D monolayer culture.

#### 4.2.2. 3D Brain Organoids

The first study describing a 3D in vitro organoid model of RTT derived from hiPSCs was performed by Mellios and colleagues [95]. In this report, the authors used an RTT hiPSC line carrying a single nucleotide substitution (missense mutation, 316C > T) in the MDB domain, a RTT hiPSC line carrying a single nucleotide deletion (frameshift 705delG) in the TRD domain, and neural progenitors derived from hiPSCs harboring a MeCP2 shRNA insert (shMeCP2), which resulted in significant reduction in MeCP2 expression. This seminal study used for the first time a 3D organoid model to test whether MeCP2 could have a regulatory impact during neurogenesis and further neuronal differentiation. hiPSC lines were first induced towards the neuroectoderm by following the dual-SMAD inhibition protocol. After 5 weeks of differentiation, brain organoids derived from the RTT cell lines revealed an increased ventricular area, with decreased ventricle wall thickness, suggesting an increased number of neural progenitors (NPs) when compared with the isogenic control. Moreover, the dendritic marker MAP2 and the early neuronal marker DCX were observed to be reduced at both protein and mRNA expression levels in RTT organoids, while PAX6, an NP marker, was enriched in the RTT-derived neural cells. More importantly, the authors also observed reduced cell migration distances for the RTT organoids, with cells mostly remaining in the PAX6^+^ proliferative/ventricular zone. Importantly, these observations were only possible due to the use of the 3D organoid system, which allowed the recapitulation and visualization of distinct self-organizing cell layers reminiscent of in vivo development of the human cortex. Moreover, in this pioneering work a reduced expression of the interneuron specification factor DLX1 was also observed. Additionally, the GABAergic interneuron marker somatostatin (STT) was also found to be reduced in RTT organoids. The authors concluded that RTT organoids showed impairments in neurogenesis and reduced dendritic complexity. Simultaneously, this RTT organoid model exhibited two up-regulated microRNAs, miR-199 and miR-214, which have an indirect role in the regulation of the ERK and AKT signaling pathways, and subsequently, in the neuronal differentiation process. This indicates that MeCP2 mutations may have a strong impact on miRNA-mediated pathways, which then impact neurogenesis and neural differentiation [95].

In a more recent work, other authors have also used a protocol for the generation of cortical organoids exhibiting two cortical regions, the MGE and the cortex, which mostly comprise GABAergic and glutamatergic neurons, respectively [83]. In this report, the authors used cortical organoids to screen the effect of the JQ1 compound. Results from single-cell RNA sequencing revealed that MeCP2-R133C region-specific organoids significantly decreased the number of dysregulated genes in both neurons and glia population, after the treatment with JQ1. Moreover, RTT organoids from the MGE region demonstrated up-regulation of genes related with forebrain development, GABAergic interneuron differentiation, and neuronal fate commitment, while in RTT cortical organoids, the JQ1 treatment improved the transcription of genes related with synaptic transmission. In fact, several clusters of different populations were analyzed, revealing, for example, that neuronal cells from RTT organoids restored the aberrant upregulation of two transcription factors, MEF2C and NEUROD2, after treatment with the drug. On the other hand, glial cells from WT-MGE organoids showed a decreased expression of transcription factors controlling immature oligodendrocytes (OL), OLIG2 and MBP, that were upregulated in the MeCP2-R133C organoids [83]. Overall, with the use of this RTT brain organoid model, it was possible to study in detail the role of MeCP2 as a transcriptional regulator in the different neural populations, over different time-points of development and across different regions of the organoids.

Finally, we have also engineered brain organoids from hiPSCs lines to recapitulate both ventral and dorsal sub-regions of the forebrain, and model RTT during neuronal development and maturation [96]. hiPSCs for organoid generation were derived from one female donor carrying the R255X mutation, and one male donor carrying the Q83X mutation. Our data revealed a premature development of the cortical subplate in the dorsal forebrain organoids developed from the female RTT hiPSC line, which was associated with an increase in the thickness of the neuronal layer and an increased expression of TBR1, a glutamatergic post-mitotic neuronal marker. The data also revealed a decrease in cell proliferation associated with the downregulation/absence of TBR2, an intermediate progenitor marker, a decreased expression of PAX6 mRNA and a lower number of HOPX oRG cells in these organoids. The previous results were consistently observed for the R255X organoids but not for the Q83X organoids, which indicates that this brain organoid model is able to highlight mutation-dependent alterations in the phenotype of RTT. Moreover, and in agreement with previously reviewed studies, the R255X dorsal organoids also revealed impairments in calcium signaling and electrophysiology analysis, with abortive-like APs, decreased AP amplitude, reduced AP velocity and absence of spontaneous synaptic transmission. In addition, the RTT dorsal neurons generated in 3D organoids, revealed a reduction in the number of mushroom and stubby shaped spines, decreased synaptogenesis and also a decrease in VGLUT1 puncta density, a specific protein of glutamatergic neurons. Furthermore, for RTT female ventral organoids, it was possible to observe a decrease of DCX expression, a marker for immature migratory interneurons. We next evaluated possible migratory impairments, a phenomenon that can be better visualized using 3D culture systems, by fusing dorsal and ventral forebrain organoids. Upon fusion, and by following the migration of GFP^+^ cells from the ventral to the dorsal side, it was possible to observe impairments in RTT GFP^+^ interneuron’s migration, with these cells migrating shorter distances than healthy GFP^+^ cells from healthy-control fused organoids.

Recent developments in the field of brain organoids have also revealed the enormous potential of this technology for personalized diagnosis and drug screening. This was the objective of Trujillo and coworkers that evaluated the effects of different pharmacological compounds in cortical organoids derived from MECP2-KO hiPSCs [88]. The 14 different drugs that were tested were first screened in 2D monolayer neuronal cultures. Based on the synaptic and neurotransmission impairments revealed by the 2D model, mainly in glutamatergic and cholinergic deregulation, and based on the hits obtained from the drug screening, two compounds, Nefiracetam, a cholinergic, GABAergic, and glutamatergic agonist, and PHA 543613, a α7-nAChR agonist with proven neuroprotective effects, were selected for further studies. After this initial screen, the authors proceeded to the use of more complex 3D brain models. They first used a neurosphere model, containing a 50/50 mixture of healthy and MeCP2-KO NPs to better mimic the mosaicism observed in female patients. After treatment with the selected compounds, the authors observed improvements in calcium transient frequency. Then, a more complex 3D system using MeCP2-KO cortical organoids was used. It was found that RTT organoids displayed smaller diameters when compared with the isogenic control. However, after 3 months of culture and 1 month of drug exposure, RTT organoids were found to increase in diameter. Moreover, RNA-seq analysis showed improvement in the expression of genes involved in synaptic function, namely neurotransmitter markers, and also an increase in neuronal spine-like protrusions, upon drug treatment. To confirm these results, functional analysis using MEA electrophysiology showed an increase in the spiking population for the RTT organoids upon drug treatment. Overall, this study revealed two promising drug candidates for future RTT clinical trials.

Overall, it is that modeling RTT with 3D brain organoid models generated from patient-specific hiPSCs and their isogenic controls, may deliver a robust understanding of patient-specific disease onset during early stages of neuronal development (Figure 2, Figure 3 and Figure 4). In addition to the studies highlighted here, there is increasing interest in the development of other region-specific brain organoids, namely midbrain, hindbrain, cerebellum, hippocampus and others, to evaluate how MeCP2 mutations affect other brain regions. Furthermore, these multiple organoid systems may also be useful for the development of personalized therapeutics and drug screening.

## 5. Future Clinical Translation of hPSC Technology in RTT

The use of hPSCs as a unique platform or combined with animal models for RTT preclinical trials is still far from being a reality. However, as we highlighted in previous sections, several hPSC-derived RTT models have been providing promising results in this field. Several drugs and the respective mechanisms of action have been studied using both hPSC-based models and animal models, which has already impacted the design of ongoing clinical trials. For example, IGF-1, which is required for proper brain development, has been tested in hiPSCs and hESC-derived neurons with several MeCP2-mutations. Interestingly, in these human RTT models, IGF-1 treatment was observed to increase the number of glutamatergic synapses [70], to rescue the soma size and dendritic complexity deficits, through the induction of AKT/mTOR activity [97]. Additionally, IGF-1 also restored KCC2 levels, by altering GABA potential [79]. In MeCP2 mutant mice, motor cortex pyramidal neurons treated with IGF-1 showed normalized spine density, synaptic amplitude and exhibited increased levels of postsynaptic density protein 95 (PSD-95), a protein present in excitatory neurons [98]. Moreover, full-length recombinant human IGF1 (rhIGF1) restored synaptic and circuit plasticity, when administrated to a MeCP2 mutant mice [99]. A phase 1 clinical trial (NCT01253317) using rhIGF-1 or Mescarmin showed safety and tolerability in girls with RTT, and even promoted improvement in breathing and behavioral abnormalities [100]. However, during phase 2 (NCT01777542), the patients assigned for rhIGF-1 did not reveal significant improvements, when compared with the placebo [101]. At the same time, another clinical trial using trofinetide (NNZ-2566), a more effective analog of IGF-1 that persists longer in the bloodstream, is already in phase 3 (started in 2020, NCT04279314). A phase 2 clinical trial for trofinetide (NCT02715115) in RTT females revealed safety, tolerability and promoted significant improvements in several symptoms, like breathing problems, mood abnormalities/disruptive behavior, neuromotor impairment, and frequency of seizures [102]. Moreover, the highest doses showed efficacy and relevant improvements of the core symptoms of RTT, such as the repetitive hand movements. Interestingly, after the cessation of the treatment, a decline in clinical advances was observed, emphasizing the consistency of the tested drug. This trial is currently recruiting patients for phase 3 (NCT04181723).

As a possible alternative, cyclic glycine-proline (cGP) has been observed to regulate the activity of IGF-1, by promoting its bioavailability when IGF-1 is insufficient [103]. Evidences demonstrated that the administration of cGP have improved synaptic expression in rats, by normalizing the expression of synaptophysin. The hypothesis behind the improvements could be associated with cGP mediation of IGF-1 bioavailability in the brain [104].

Moreover, a female patient with the missense mutation, R106W, showed a decreased symptomatology after the 6 months treatment with IGF-I, melatonin and also blackcurrant extracts, which contain cGP [105]. Interestingly, melatonin is a neurohormone that acts as a potent antioxidant, regarding its role as a free radical scavenger [106]. Scientific evidence suggested increased oxidative stress in MeCP2 mutant neurons [107], potentially due to functional and structural mitochondrial alterations [108]. Within further studies, melatonin could be a potential candidate drug for a translational approach, due to its known function and since it has been also been tested in patients in neurodevelopmental disorders [109].

Over the past few years, gene therapy approaches were also developed for RTT treatment, namely through the use of adeno-associated viral (AAV) vectors, such as AAV9, to mediate *MeCP2* gene transfer or gene editing [110]. In fact, AAVs were shown to be the best vehicles in CNS, due to its tropism and capacity to cross the blood–brain barrier (BBB) [110]. Two major concerns regarding this potential therapeutic approach still persist, particularly viral toxicity and heterogenous distribution of *MeCP2* gene expression throughout the brain, since local overexpression causes severe symptoms, as observed in MeCP2 duplication syndrome [111]. Recently, another publication showed that AAV9 coupled with the use of CRISPR/Cas9 was able to target and correct FOXG1 in both RTT hiPSCs and hiPSC-derived neurons, with an efficiency of 20–35% [112]. Very close to clinical trials is also an AAV-mediated *MeCP2* gene expression cassette [113] with endogenous regulatory elements, already tested in pre-clinical mouse models [114]. This study revealed improvement in RTT-like phenotype without apparent toxicity [114,115]. More specifically, the system consists of an *MeCP2* expression cassette with a modified endogenous *MeCP2* promoter to limit transcription and 3′ UTR with binding sites for microRNAs (miRNAs) that are known to regulate *MeCP2* expression [114,115,116,117]. This control system will allow the safety of the transgene expression levels in brain cells. A phase 1 clinical trial is expected to be initiated soon, with the objective of assessing the safety and tolerability of this vector system.

A great number of preclinical studies has been relying on mouse models. However, despite the general conservation among different mammal species, there are several characteristics that are absent in rodent models, particularly human-specific gene expression, neurotransmission and cell layers during human cortical development. For example, rodents lack the proliferative outer radial glia cells (oRGCs) in the outer subventricular zone (oSVZ), a hallmark of human brain evolution during development [118]. The glutamatergic, serotoninergic, and cholinergic systems have also been observed to be different between humans and rodents [119]. Some of the previous evidence could somehow explain the failure in late-stage clinical trials, mainly during phases 2 and 3. Unfortunately, a good example is a recently finished clinical trial with Sarizotan, an agonist of the serotonergic 5HT1A receptor designed to bind to neurotransmitters, such as dopamine and serotonin. Phase 2 and 3 clinical trials (NCT02790034) did not demonstrate evidence of efficacy when compared with the placebo group, in targeting respiratory symptoms in RTT patients, and also failed in other secondary objectives, like effectiveness and improvements in motor function [120]. The study was designed based on data extracted from mouse models (MeCP2 null male and heterozygous females, one with a common nonsense mutation (R168X), and the other with deletions in exon 3 and 4), being observed a decrease in apnea incidence of 25–33% [121]. This example highlights the inherent difficulty in translating clinical effects observed in animal models into humans and the need for optimization using human brain models, such as models derived from patient-specific hiPSCs, in preclinical stages [122]. In fact, interesting drug candidates validated in hPSCs-based RTT models could be selected for further consideration, for example, Nefiracetam and PHA 543613 [88], Gentamicin [70], JQ1 [83], Choline [76], Rolipram [77] or inhibitors of HDAC6 [82].

## 6. hPSC-Derived RTT Brain Models-Limitations and Future Directions

The number and relevance of RTT in vitro cellular models, especially the ones generated from hPSCs, has greatly increased during the last decade. From 2D to 3D platforms, RTT has been intensively studied using hPSC-derived models with several neurobiological questions under consideration. Overall, both model formats are appealing but they also have several limitations that current and future methodologies are trying to overcome.

For disease modeling, neuronal cell cultures differentiated under 2D conditions were revealed to be more homogeneous, resembling neurons from specific brain regions. In fact, these culture systems provide an homogeneous distribution of nutrients and small-molecules [123]. However, the patterning process is not accompanied by the proper formation of distinct cell layers, missing the presence of relevant types of neural progenitors, neurons or non-neural cells. Moreover, synaptic connection and network formation across specialized neural cell types, which is the basis for proper brain function, does not occur efficiently [124]. The development of 3D organoids overcame almost all these disadvantages while allowing a closer mimic of the in vivo steps of human fetal neurodevelopment and maturation [89]. Nevertheless, 3D brain organoid models are not yet fully optimized for translational applications, creating the need to develop reproducible culture conditions alongside more advanced tissue engineering strategies [125]. The proper diffusion of oxygen, nutrients and small molecules may be compromised due to the dimensions of the organoids in culture and by the lack of vascularization, increasing the necrosis at the core [126]. Moreover, batch-to-batch variability is intrinsic to these 3D culture systems, creating a variable number of cortical zones. Some strategies have been implemented to overcome these limitations. Benefiting from the knowledge of neurodevelopmental biology principles, directed differentiation processes using the appropriate growth factors and small molecules have been able to induce the generation of region-specific organoids, decreasing inter-organoid variability [125]. Several methodologies have been able to generate organoids resembling, for example, the cerebral cortex [127,128], the ventral forebrain [91,129], the cerebellum [130,131,132], the midbrain with functional dopaminergic neurons [133,134] and the spinal cord [135]. Moreover, the fusion of different regional organoids [91,136] can be used to model the kinetic processes of cell migration, cell–cell interactions and neural circuit formation. Importantly, a bioengineering strategy that has been proved to ameliorate diffusional problems and decrease organoids heterogeneity, rely on the use of diverse stirred bioreactor systems for brain organoid development [90,137,138]. In such dynamic systems, 3D organoids could be maintained in culture for prolonged culture periods, achieving important maturation milestones like the formation of important regulatory populations, such as astrocytes, microglia, and oligodendrocytes [137]. Moreover, recently described protocols disclosed the generation of tube-like vascular systems in brain organoids, by co-culture with human umbilical vein endothelial cells (HUVECs) [139], or for example, by using a 3D printing-based platform to co-culture organoids with hiPSC-derived pericytes and endothelial cells, which were capable of organizing into vascular networks [140].

Definitely, another important consideration when modeling RTT relies on the importance of using the proper isogenic control since this greatly increases the confidence in the results, excluding differences generated by the genetic background of the donors. In RTT hiPSCs, the location of the mutation in the X chromosome and the XCI process are two advantages that contribute for the straightforward generation of isogenic controls. Therefore, the strategy of mixing both mutated and non-mutated hiPSCs for further generation of mosaic brain organoids, [88], or the use of each patient own cells, with their specific mutation and background, makes brain organoids even closer to becoming clinically relevant in RTT.

In summary, the rapid progression in advanced tissue engineering strategies and brain organoid systems are closer to recreate brain genetic and functional environments, with the capability of being used as humanized models in a disease context. The realistic complexity that has been achieved in brain organoid systems should thus be capable of properly modeling RTT. When combined with a high throughput analysis, such as RNA-sequencing, proteomics and metabolomics, and functional analysis, these technologies will more faithfully be allowed to recapitulate and to characterize RTT developmental features. These more complex systems are becoming closer to clinical translation by allowing drug discovery and evaluation of patient-specific drug responses.

## Data Availability

Not applicable.

## Abbreviations

ASD	autism spectrum disorder
AD	Alzheimer’s disease
AMPA	2-Amino-3-(3-hydroxy-5-methyl-isoxazol-4-yl) propanoic acid
AAV	adeno-associated virus
BBB	blood brain barrier
BDNF	brain-derived neurotrophic factor
BRD4	bromodomain 4
CDKL5	cyclin dependent kinase like 5
CNS	central nervous system
CTD	C-terminal domain
EB	embryoid bodies
E/I	excitatory/inhibitory imbalance
EVs	extracellular vesicles
FOXG1	forkhead box G1
FLT3	fms-like tyrosine kinase 3
GABA	gamma-aminobutyric acid
GFAP	glial fibrillary acidic protein
GluR2/3	glutamate receptor subunit
HDAC	histone deacetylases
HDAC6	histone deacetylases 6
hESCs	human embryonic stem cells
hiPSCs	human induced pluripotent stem cells
Hsp	heat-shock proteins
HTS	high-throughput screening
HUVECs	human umbilical vein endothelial cells
IC	isogenic control
ID	intervening domain
IGF-1	insulin-like growth factor-1
IL	interleukin
ILVs	intraluminal vesicles
INs	interneurons
L1CAM L1	cell adhesion molecule
LPS	lipopolysaccharides
MBD	methyl-CpG binding domain
MEA	multi electrode array
MeCP2	methyl-CpG-binding protein 2
MeCP2LOF	methyl-CpG-binding protein 2 loss of function
MeCP2-KO	methyl-CpG-binding protein 2 knocking out
MGE	medial ganglionic eminence
miRNAs	micro RNAs
mRNAs	messenger RNA
mtDNA	mitochondrial DNA
MVs	microvesicles
MVBs	multivesicular bodies
NCOR	nuclear co-repressor
NMDA	N-methyl-d-aspartate
NPs	neural progenitors
NPCs	neural progenitor cells
NTD	N-terminal domain
OL	oligodendrocytes
oRGCs	outer radial glia cells
oSVZ	outer subventricular zone
PSD-95	postsynaptic density protein 95
RG	radial glia
rhIGF1	full-length recombinant human IGF1
RNA	ribonucleic acid
RNA-seq	ribonucleic acid sequencing
ROS	reactive oxygen species
RTT	Rett Syndrome
siRNA	small interfering RNA
SMRT	silencing mediator of retinoic acid and thyroid hormone receptor
STT	somatostatin
TGF-β1	transforming growth factor beta 1
TRD	transcriptional repression domain
TrkB	tropomyosin-related kinase B
KCC2	neuron-specific K^+^-Cl^−^ cotransporter 2
XCI	X-chromosome inactivation

## References

[1] Reichow B., Tara A.G., Isaac L., Volkmar F.R., Boy M.Á. (2015). Brief Report: Systematic Review of Rett Syndrome in Males. J. Autism Dev. Disord..

[2] Chahrour M., Zoghbi H.Y. (2007). The Story of Rett Syndrome: From Clinic to Neurobiology. Neuron.

[3] Wan M., Sung S., Lee J., Zhang X., Houwink-manville I., Song H., Amir R.E., Budden S., Naidu S., Pereira J.L.P. (1999). Rett Syndrome and Beyond: Recurrent Spontaneous and Familial MECP2 Mutations at CpG Hotspots. Am. J. Hum. Genet..

[4] Martínez De Paz A., Khajavi L., Martin H., Claveria-Gimeno R., Tom Dieck S., Cheema M.S., Sanchez-Mut J.V., Moksa M.M., Carles A., Brodie N.I. (2019). MeCP2-E1 isoform is a dynamically expressed, weakly DNA-bound protein with different protein and DNA interactions compared to MeCP2-E2. Epigenetics Chromatin.

[5] Cheadle J.P., Gill H., Fleming N., Maynard J., Kerr A., Leonard H., Krawczak M., Cooper D.N., Lynch S., Thomas N. (2000). Long-read sequence analysis of the MECP2 gene in Rett syndrome patients: Correlation of disease severity with mutation type and location. Hum. Mol. Genet..

[6] Neul J.L., Fang P., Barrish J., Lane J., Caeg E.B., Smith E.O., Zoghbi H., Percy A., Glaze D.G. (2008). Specific mutations in Methyl-CpG-Binding Protein 2 confer different severity in Rett syndrome. Neurology.

[7] Caffarelli C., Gonnelli S., Pitinca M.D.T., Camarri S., Al Refaie A., Hayek J., Nuti R. (2020). Methyl-CpG-binding protein 2 (MECP2) mutation type is associated with bone disease severity in Rett syndrome. BMC Med. Genet..

[8] Cuddapah V.A., Pillai R.B., Shekar K.V., Lane J.B., Motil K.J., Skinner S.A., Tarquinio D.C., Glaze D.G., McGwin G., Kaufmann W.E. (2014). Methyl-CpG-binding protein 2 (MECP2) mutation type is associated with disease severity in rett syndrome. J. Med. Genet..

[9] Hoffbuhr K.C., Moses L.M., Jerdonek M.A., Naidu S., Hoffman E.P. (2002). Associations between MeCP2 mutations, X-chromosome inactivation, and phenotype. Ment. Retard. Dev. Disabil. Res. Rev..

[10] Ishii T., Makita Y., Ogawa A., Amamiya S., Yamamoto M. (2001). The role of different X-inactivation pattern on the variable clinical phenotype with Rett syndrome. Brain Dev..

[11] Zoghbi H.Y., Percy A.K., Schultz R.J., Fill C. (1990). Patterns of X Chromosome Inactivation in the Rett Syndrome. Brain Dev..

[12] Shahbazian M.D., Sun Y., Zoghbi H.Y. (2002). Balanced X chromosome inactivation patterns in the Rett syndrome brain. Am. J. Med. Genet..

[13] Ribeiro M.C., Macdonald J.L. (2020). Sex differences in Mecp2 -mutant Rett syndrome model mice and the impact of cellular mosaicism in phenotype development. Brain Res..

[14] Wochenschr W.M. (1996). On a unusual brain atrophy syndrome in hyperammonemia in childhood. Wien. Med. Wochenschr..

[15] Amir R.E., Van Den Veyver I.B., Wan M., Tran C.Q., Francke U., Zoghbi H.Y. (1999). Rett syndrome is caused by mutations in X-linked MECP2, encoding methyl-CpG-binding protein 2. Nature.

[16] Evans J.C., Archer H.L., Colley J.P., Ravn K., Nielsen J.B., Kerr A., Williams E., Christodoulou J., Jardine P.E., Wright M.J. (2005). Early onset seizures and Rett-like features associated with mutations in CDKL5. Eur. J. Hum. Genet..

[17] Philippe C., Amsallem D., Francannet C., Lambert L., Saunier A., Verneau F., Jonveaux P. (2010). Phenotypic variability in Rett syndrome associated with FOXG1 mutations in females. Med. Genet..

[18] Cosentino L., Vigli D., Franchi F., Laviola G., Filippis B. (2019). De Rett syndrome before regression: A time window of overlooked opportunities for diagnosis and intervention. Neurosci. Biobehav. Rev..

[19] Halbach N., Smeets E., Steinbusch C., Maaskant M., van Waardenburg D., Curfs L. (2013). Aging in Rett syndrome: A longitudinal study. Clin. Genet..

[20] Killian J.T., Lane J.B., Lee H.-S., Skinner S.A., Kaufmann W.E., Glaze D.G., Neul J.L. (2017). Scoliosis in Rett Syndrome: Progression, Comorbidities, and Predictors. Pediatr. Neurol..

[21] Roze E., Sangla S., Bienvenu T., Leu-semenescu S. (2007). Rett Syndrome: An Overlooked Diagnosis in Women with Stereotypic Hand Movements, Psychomotor Retardation, Parkinsonism, and Dystonia?. Mov. Disord..

[22] Tarquinio D.C., Hou W., Neul J.L., Kaufmann W.E., Glaze D.G., Motil K.J., Skinner S.A., Lee H., Percy A.K. (2015). The Changing Face of Survival in Rett Syndrome and MECP2 -Related Disorders. Pediatr. Neurol..

[23] Huppke P., Laccone F., Krämer N., Engel W., Hanefeld F. (2000). Rett syndrome: Analysis of MECP2 and clinical characterization of 31 patients. Hum. Mol. Genet..

[24] Murakami J.W., Courchesne E., Haas R.H., Press G.A., Yeung-courchesne R. (1992). Cerebellar and Cerebral Abnormalities in Rett Syndrome: A Quantitative MR Analysis. Ajr. Am. J. Roentgenol..

[25] Cardoza B., Clarke A., Wilcox J., Gibbon F., Smith P.E.M., Archer H., Hryniewiecka-jaworska A., Kerr M. (2011). Epilepsy in Rett syndrome: Association between phenotype and genotype, and implications for practice. Seizure.

[26] Glaze D.G., Percy A.K., Skinner S., Motil K.J., Neul J.L., Barrish O., Lane J.B., Geerts S.P., Annese F., Graham J. (2010). Epilepsy and the natural history of Rett syndrome. Neurology.

[27] Nectoux J., Bahi-Buisson N., Guellec I., Coste J., De Roux N., Rosas H., Tardieu M., Chelly J., Bienvenu T. (2008). The p. Val66Met polymorphism in the BDNF gene protects against early seizures in Rett syndrome. Neurology.

[28] Ben Zeev B., Leonard H., De Klerk N. (2009). The common BDNF polymorphism may be a modifier of disease severity in Rett syndrome. Neurology.

[29] Buoni S., Zannolli R., De Felice C., Saponari S., Strambi M., Teresa M., Castrucci E., Corbini L., Orsi A., Hayek J. (2008). Drug-resistant epilepsy and epileptic phenotype-EEG association in MECP2 mutated Rett syndrome. Clin. Neurophysiol..

[30] Krajnc N. (2015). Management of epilepsy in patients with Rett syndrome: Perspectives and considerations. Clin. Risk Manag..

[31] Geerdink N., Rotteveel J.J., Lammens M., Sistermans E.A., Heikens G.T., Mullaart R.A., Hamel B.C.J. (2002). MECP2 Mutation in a Boy with Severe Neonatal Encephalopathy: Clinical, Neuropathological and Molecular Findings. Neuropediatrics.

[32] Bourdon V., Philippe C., Martin D., Verlo A., Jonveaux P. (2003). MECP2 Mutations or Polymorphisms in Mentally Retarded Boys. Mol. Diagn..

[33] Neul J.L., Benke T.A., Marsh E.D., Skinner S.A., Merritt J., Lieberman D.N., Standridge S., Feyma T., Heydemann P., Peters S. (2019). The array of clinical phenotypes of males with mutations in Methyl-CpG binding protein 2. Am. J. Med. Genet..

[34] Schwartzman J.S., Bernardino A., Nishimura A., Gomes R.R., Zatz M., Mackenzie U., Paulo S. (2001). Rett Syndrome in a Boy with a 47, XXY Karyotype Confirmed by a Rare Mutation in the MECP2 Gene. Neuropediatrics.

[35] Zhang Q., Yang X., Wang J., Li J., Wu Q. (2019). Genomic mosaicism in the pathogenesis and inheritance of a Rett syndrome cohort. Genet. Med..

[36] Pieras J.I., Mun B., Borrego S., Marcos I., Sanchez J., Madruga M., Antin G. (2011). Somatic mosaicism for Y120X mutation in the MECP2 gene causes atypical Rett syndrome in a male. Brain Dev..

[37] Van Esch H., Bauters M., Ignatius J., Jansen M., Raynaud M., Hollanders K., Lugtenberg D., Bienvenu T., Jensen L.R., Ge J. (2005). Duplication of the MECP2 Region Is a Frequent Cause of Severe Mental Retardation and Progressive Neurological Symptoms in Males. Am. J. Hum. Genet..

[38] Gaudio D., Fang P., Scaglia F., Ward P.A., Craigen W.J. (2006). Increased MECP2 gene copy number as the result of genomic duplication in neurodevelopmentally delayed males. Genet. Med..

[39] Miguet M., Faivre L., Amiel J., Nizon M., Touraine R., Prieur F., Pasquier L., Lefebvre M., Thevenon J., Dubourg C. (2018). Further delineation of the MECP2 duplication syndrome phenotype in 59 French male patients, with a particular focus on morphological and neurological features. J. Med. Genet..

[40] Yu B., Yuan B., Dai J.K., Cheng T.-l., Xia S.N., He L.J., Yuan Y.T., Zhang Y.F., Xu H.T., Xu F.Q. (2020). Reversal of Social Recognition Deficit in Adult Mice with MECP2 Duplication via Normalization of MeCP2 in the Medial Prefrontal Cortex. Neurosci. Bull..

[41] Nageshappa S., Carromeu C., Trujillo C.A., Mesci P., Espuny-Camacho I., Pasciuto E., Vanderhaeghen P., Verfaillie C.M., Raitano S., Kumar A. (2016). Altered neuronal network and rescue in a human MECP2 duplication model. Mol. Psychiatry.

[42] Liyanage V.R.B., Rastegar M., Mecp M.Á.M.Á., Rett Á. (2014). Rett Syndrome and MeCP2. Neuromol. Med.

[43] Ehrhart F., Coort S.L.M., Cirillo E., Smeets E., Evelo C.T., Curfs L.M.G. (2016). Rett syndrome—Biological pathways leading from MECP2 to disorder phenotypes. Orphanet J. Rare Dis..

[44] Horvath P.M., Monteggia L.M. (2019). MeCP2 as an activator of gene expression. Trends Neurosci..

[45] Picard N., Fagiolini M. (2019). MeCP2: An epigenetic regulator of critical periods. Curr. Opin. Neurobiol..

[46] Tillotson R., Bird A. (2020). The Molecular Basis of MeCP2 Function in the Brain. J. Mol. Biol..

[47] Pejhan S., Rastegar M. (2021). Role of dna methyl-cpg-binding protein mecp2 in rett syndrome pathobiology and mechanism of disease. Biomolecules.

[48] Nan X., Meehan R.R., Bird A. (1993). Dissection of the methyl-CpG binding domain from the chromosomal protein MeCP2. Nucleic Acids Res..

[49] Nan X., Ng H., Johnson C.A., Laherty C.D., Turner B.M., Eisenman R.N., Bird A. (1998). Transcriptional repression by the methyl-CpG-binding protein MeCP2 involves a histone deacetylase complex. Lett. Nat..

[50] Lagger S., Connelly J.C., Schweikert G., Webb S., Selfridge J., Ramsahoye B.H., Yu M., He C., Sanguinetti G., Sowers L.C. (2017). MeCP2 recognizes cytosine methylated tri- nucleotide and di-nucleotide sequences to tune transcription in the mammalian brain. PLoS Genet..

[51] Kokura K., Kaul S.C., Wadhwa R., Nomura T., Khan M., Shinagawa T., Yasukawa T., Colmenares C., Ishii S. (2001). The Ski Protein Family Is Required for MeCP2-mediated Transcriptional Repression. J. Biol. Chem..

[52] Lyst M.J., Ekiert R., Ebert D.H., Merusi C., Nowak J., Selfridge J., Guy J., Kastan N.R., Robinson N.D., Alves F.D.L. (2013). Rett syndrome mutations abolish the interaction of MeCP2 with the NCoR/SMRT co-repressor. Nat. Neurosci..

[53] Boxer L.D., Renthal W., Greben A.W., Griffith E.C., Bonev B., Greenberg M.E., Boxer L.D., Renthal W., Greben A.W., Whitwam T. (2020). MeCP2 Represses the Rate of Transcriptional Initiation of Highly Methylated Long Genes. Mol. Cell.

[54] Chen L., Chen K., Lavery L.A., Andrew S., Shaw C.A., Li W. (2015). MeCP2 binds to non-CG methylated DNA as neurons mature, influencing transcription and the timing of onset for Rett syndrome. Proc. Natl. Acad. Sci. USA.

[55] Kinde B., Gabel H.W., Gilbert C.S., Griffith E.C., Greenberg M.E. (2015). Reading the unique DNA methylation landscape of the and MeCP2. Proc. Natl. Acad. Sci. USA.

[56] Gabel H.W., Kinde B., Stroud H., Gilbert C.S., Harmin D.A., Kastan N.R., Hemberg M., Ebert D.H., Greenberg M.E. (2015). Disruption of DNA-methylation-dependent long gene repression in Rett syndrome. Nature.

[57] Clemens A.W., Wu D.Y., Moore J.R., Christian D.L., Zhao G., Gabel H.W., Methylation C.T.D.N.A., Clemens A.W., Wu D.Y., Moore J.R. (2020). MeCP2 Represses Enhancers through Chromosome Article MeCP2 Represses Enhancers through. Mol. Cell.

[58] Chahrour M., Jung S.Y., Shaw C., Zhou X., Wong S.T.C., Zoghbi H.Y. (2008). MeCP2, a Key Contributor to Neurological Disease, Activates and Represses Transcription. Science.

[59] Ito-ishida A., Baker S.A., Sillitoe R.V., Sun Y., Zhou J., Ono Y., Iwakiri J., Yuzaki M., Zoghbi H.Y. (2020). MeCP2 Levels Regulate the 3D Structure of Heterochromatic Foci in Mouse Neurons. J. Neurosci..

[60] Baker S.A., Chen L., Wilkins A.D., Yu P., Lichtarge O., Zoghbi H.Y. (2013). An AT-Hook Domain in MeCP2 Determines the Clinical Course of Rett Syndrome and Related Disorders. Cell.

[61] Squillaro T., Hayek G., Farina E., Cipollaro M., Renieri A., Galderisi U. (2008). A case report: Bone marrow mesenchymal stem cells from a rett syndrome patient are prone to senescence and show a lower degree of apoptosis. J. Cell. Biochem..

[62] Squillaro T., Alessio N., Capasso S., Di Bernardo G., Melone M.A.B., Peluso G., Galderisi U. (2019). Senescence phenomena and metabolic alteration in mesenchymal stromal cells from a mouse model of rett syndrome. Int. J. Mol. Sci..

[63] Ip J.P.K., Mellios N., Sur M. (2018). Rett syndrome: Insights into genetic, molecular and circuit mechanisms. Nat. Rev. Neurosci..

[64] Colantuoni C., Jeon O.H., Hyder K., Chenchik A., Khimani A.H., Narayanan V., Hoffman E.P., Kaufmann W.E., Naidu S.B., Pevsner J. (2001). Gene expression profiling in postmortem Rett Syndrome brain: Differential gene expression and patient classification. Neurobiol. Dis..

[65] Zeng R., Sidik H., Robinson K.S., Zhong F.L., Reversade B. (2019). Generation of four H1 hESC sublines carrying a hemizygous knock-out/mutant MECP2. Stem Cell Res..

[66] Thanh T., Le H., Tran N.T., Mai T., Dao L., Nguyen D.D., Do H.D., Ha T.L., Kühn R., Nguyen T.L. (2019). Efficient and Precise CRISPR/Cas9- Mediated MECP2 Modifications in Human-Induced Pluripotent Stem Cells. Front. Genet..

[67] Tchieu J., Kuoy E., Chin M.H., Trinh H., Patterson M., Sean P., Aimiuwu O., Lindgren A., Hakimian S., Zack J.A. (2010). Female human iPS cells retain inactive X-chromosome. Cell Stem Cell.

[68] Cheung A.Y.L., Horvath L.M., Carrel L., Ellis J., Colman A. (2012). X-chromosome inactivation in Rett syndrome human induced pluripotent stem cells. Front. Psychiatry.

[69] Bar S., Seaton L.R., Weissbein U., Eldar-geva T., Bar S., Seaton L.R., Weissbein U., Eldar-geva T., Benvenisty N. (2019). Global Characterization of X Chromosome Report Global Characterization of X Chromosome Inactivation in Human Pluripotent Stem Cells. Cell Rep..

[70] Marchetto M.C.N., Carromeu C., Acab A., Yu D., Yeo G.W., Mu Y., Chen G., Gage F.H., Muotri A.R. (2010). A Model for Neural Development and Treatment of Rett Syndrome Using Human Induced Pluripotent Stem Cells. Cell.

[71] Ananiev G., Williams E.C., Li H., Chang Q. (2011). Isogenic Pairs of Wild Type and Mutant Induced Pluripotent Stem Cell ( iPSC ) Lines from Rett Syndrome Patients as In Vitro Disease Model. PLoS ONE.

[72] Kim K.Y., Hysolli E., Park I.H. (2011). Neuronal maturation defect in induced pluripotent stem cells from patients with Rett syndrome. Proc. Natl. Acad. Sci. USA.

[73] Djuric U., Cheung A.Y.L., Zhang W., Mok R.S., Piekna A., Hendry J.A., Ross P.J., Pasceri P., Kim D., Salter M.W. (2015). MECP2e1 isoform mutation affects the form and function of neurons from Rett syndrome patient iPS cells. Neurobiol. Dis..

[74] Chambers S.M., Fasano C.A., Papapetrou E.P., Tomishima M., Sadelain M., Studer L. (2009). Highly efficient neural conversion of human ES and iPS cells by dual inhibition of SMAD signaling. Nat. Biotechnol..

[75] Fernandes T.G., Duarte S.T., Ghazvini M., Gaspar C., Santos D.C. (2015). Neural commitment of human pluripotent stem cells under defined conditions recapitulates neural development and generates patient-specific neural cells. Biotechnol. J..

[76] Chin E.W.M., Marcy G., Yoon S.I., Ma D., Rosales F.J., Augustine G.J., Goh E.L.K. (2016). Choline Ameliorates Disease Phenotypes in Human iPSC Models of Rett Syndrome. Neuromol. Med..

[77] Bu Q., Wang A., Hamzah H., Waldman A., Jiang K., Dong Q., Li R., Kim J., Turner D., Chang Q. (2017). CREB signaling is involved in rett syndrome pathogenesis. J. Neurosci..

[78] Yoo M., Carromeu C., Kwon O., Muotri A., Schachner M. (2017). The L1 adhesion molecule normalizes neuritogenesis in Rett syndrome-derived neural precursor cells. Biochem. Biophys. Res. Commun..

[79] Tang X., Kim J., Zhou L., Wengert E., Zhang L., Wu Z., Carromeu C., Muotri A.R. (2015). KCC2 rescues functional deficits in human neurons derived from patients with Rett syndrome. Proc. Natl. Acad. Sci. USA.

[80] Tang X., Drotar J., Li K., Clairmont C.D., Brumm A.S., Sullins A.J., Wu H., Liu X.S., Wang J., Gray N.S. (2019). Pharmacological enhancement of KCC2 gene expression exerts therapeutic effects on human Rett syndrome neurons and Mecp2 mutant mice. Sci. Transl. Med..

[81] Ohashi M., Korsakova E., Allen D., Lee P., Fu K., Vargas B.S., Cinkornpumin J., Salas C., Park J.C., Germanguz I. (2018). Loss of MECP2 Leads to Activation of P53 and Neuronal Senescence. Stem Cell Rep..

[82] Landucci E., Brindisi M., Bianciardi L., Catania L.M., Daga S., Croci S., Frullanti E., Fallerini C., Butini S., Brogi S. (2018). iPSC-derived neurons profi ling reveals GABAergic circuit disruption and acetylated α -tubulin defect which improves after iHDAC6 treatment in Rett syndrome. Exp. Cell Res..

[83] Xiang Y., Tanaka Y., Patterson B., Sullivan G.J., Weissman S.M., Park I., Xiang Y., Tanaka Y., Patterson B., Hwang S. (2020). Dysregulation of BRD4 Function Underlies the Functional Abnormalities of MeCP2 Mutant Neurons. Mol. Cell.

[84] Varderidou-minasian S., Hinz L., Hagemans D., Posthuma D., Altelaar M., Heine V.M. (2020). Quantitative proteomic analysis of Rett iPSC-derived neuronal progenitors. Mol. Autism.

[85] Sharma P., Mesci P., Carromeu C., McClatchy D.R., Schiapparelli L., Yates J.R., Muotri A.R., Cline H.T. (2019). Exosomes regulate neurogenesis and circuit assembly. Proc. Natl. Acad. Sci. USA.

[86] Chen X., Han X., Blanchi B., Guan W., Ge W., Yu Y., Sun Y.E. (2020). Graded and pan-neural disease phenotypes of Rett Syndrome linked with dosage of functional MeCP2. Protein Cell.

[87] Benito-kwiecinski S., Lancaster M.A. (2020). Brain Organoids: Human Neurodevelopment in a Dish. Cold Spring Harb. Perspect. Biol..

[88] Trujillo C.A., Adams J.W., Negraes P.D., Carromeu C., Tejwani L., Acab A., Tsuda B., Thomas C.A., Sodhi N., Fichter K.M. (2020). Pharmacological reversal of synaptic and network pathology in human MECP 2 -KO neurons and cortical organoids. EMBO Mol. Med..

[89] Lancaster M.A., Renner M., Martin C., Wenzel D., Bicknell L.S., Hurles M.E., Homfray T., Penninger J.M., Jackson A.P., Knoblich J.A. (2013). Cerebral organoids model human brain development and microcephaly. Nature.

[90] Qian X., Nguyen H.N., Song M.M., Hadiono C., Ogden S.C., Hammack C., Yao B., Hamersky G.R., Jacob F., Zhong C. (2016). Brain-Region-Specific Organoids Using Mini- bioreactors for Modeling ZIKV Exposure. Cell.

[91] Bagley J.A., Reumann D., Bian S., Lévi-strauss J., Knoblich J.A. (2017). Fused cerebral organoids model interactions between brain regions. Nat. Methods.

[92] Qi Y., Zhang X., Renier N., Wu Z., Atkin T., Sun Z., Ozair M.Z., Tchieu J., Zimmer B., Fattahi F. (2017). Combined small-molecule inhibition accelerates the derivation of functional cortical neurons from human pluripotent stem cells. Nat. Biotechnol..

[93] Zhang Z.N., Freitas B.C., Qian H., Lux J., Acab A., Trujillo C.A., Herai R.H., Huu V.A.N., Wen J.H., Joshi-Barr S. (2016). Layered hydrogels accelerate iPSC-derived neuronal maturation and reveal migration defects caused by MeCP2 dysfunction. Proc. Natl. Acad. Sci. USA.

[94] Nguyen A.T., Mattiassi S., Loeblein M., Chin E., Ma D., Coquet P., Viasnoff V., Teo E.H.T., Goh E.L., Yim E.K.F. (2018). Human Rett-derived neuronal progenitor cells in 3D graphene scaffold as an in vitro platform to study the effect of electrical stimulation on neuronal differentiation. Biomed. Mater..

[95] Mellios N., Feldman D.A., Sheridan S.D., Ip J.P.K., Kwok S., Amoah S.K., Rosen B., Rodriguez B.A., Crawford B., Swaminathan R. (2018). MeCP2-regulated miRNAs control early human neurogenesis through differential effects on ERK and AKT signaling. Mol. Psychiatry.

[96] Gomes A.R., Fernandes T.G., Vaz S.H., Silva T.P., Bekman E.P., Xapelli S., Duarte S., Ghazvini M., Gribnau7 J., Muotri A.R. (2020). Modeling Rett Syndrome With Human Patient-Specific Forebrain Organoids. Front. Cell Dev. Biol..

[97] Kwok S., Li Y., Wang H., Muffat J., Cheng A.W., Orlando D.A., Love J., Feldman D.A., Bateup H.S., Gao Q. (2013). Global Transcriptional and Translational Repression in Human-Embryonic-Stem-Cell-Derived Rett Syndrome Neurons. Cell Stem Cell..

[98] Tropea D., Giacometti E., Wilson N.R., Beard C., Mccurry C., Dong D., Flannery R., Jaenisch R., Sur M. (2009). Partial reversal of Rett Syndrome-like symptoms in MeCP2 mutant mice. Proc. Natl. Acad. Sci. USA.

[99] Castro J., Garcia R.I., Kwok S., Banerjee A., Petravicz J., Woodson J. (2014). Functional recovery with recombinant human IGF1 treatment in a mouse model of Rett Syndrome. Proc. Natl. Acad. Sci. USA.

[100] Khwaja O.S., Ho E., Barnes K.V., Leary H.M.O., Pereira L.M., Finkelstein Y. (2014). Safety, pharmacokinetics, and preliminary assessment of efficacy of mecasermin (recombinant human IGF-1) for the treatment of Rett syndrome. Proc. Natl. Acad. Sci. USA.

[101] Leary H.M.O., Kaufmann W.E., Barnes K.V., Rakesh K., Kapur K., Tarquinio D.C., Cantwell N.G., Roche K.J., Rose S.A., Alexandra C. (2018). Placebo-controlled crossover assessment of mecasermin for the treatment of Rett syndrome. Neurology.

[102] Glaze D.G., Neul J.L., Kaufmann W.E., Berry-kravis E. (2019). Double-blind, randomized, placebo-controlled study of trofinetide in pediatric Rett syndrome. Neurology.

[103] Guan J., Gluckman P., Yang P., Krissansen G., Sun X., Zhou Y., Wen J., Phillips G., Shorten P.R., McMahon C.D. (2014). Cyclic glycine-proline regulates IGF-1 homeostasis by altering the binding of IGFBP-3 to IGF-1. Sci. Rep..

[104] Li F., Liu K., Wang A., Harris P.W.R., Vickers M.H., Guan J. (2019). Cyclic glycine-proline administration normalizes high-fat diet-induced synaptophysin expression in obese rats. Neuropeptides.

[105] Devesa J., Devesa O., Carrillo M., Casteleiro N., Devesa A., Llorente D., González C. (2018). Rett Syndrome: Treatment with IGF-I, Melatonin, Blackcurrant Extracts, and Rehabilitation. Reports.

[106] Sánchez A., Calpena A.C., Clares B. (2015). Evaluating the oxidative stress in inflammation: Role of melatonin. Int. J. Mol. Sci..

[107] De Felice C., Della Ragione F., Signorini C., Leoncini S., Pecorelli A., Ciccoli L., Scalabrì F., Marracino F., Madonna M., Belmonte G. (2014). Oxidative brain damage in Mecp2-mutant murine models of Rett syndrome. Neurobiol. Dis..

[108] Shulyakova N., Andreazza A.C., Mills L.R., Eubanks J.H. (2017). Mitochondrial dysfunction in the pathogenesis of rett syndrome: Implications for mitochondria-targeted therapies. Front. Cell. Neurosci..

[109] De Leersnyder H., Zisapel N., Laudon M. (2011). Prolonged-release melatonin for children with neurodevelopmental disorders. Pediatr. Neurol..

[110] Lykken E.A., Shyng C., Edwards R.J., Rozenberg A., Gray S.J. (2018). Recent progress and considerations for AAV gene therapies targeting the central nervous system. J. Neurodev. Disord..

[111] Katz D.M., Bird A., Coenraads M., Gray S.J., Menon D.U., Philpot B.D., Tarquinio D.C. (2016). Rett Syndrome: Crossing the Threshold to Clinical Translation. Trends Neurosci..

[112] Croci S., Lucia M., Katia C., Daga S., Donati F., Tiziana F., Elisa P., Diego F., Lamacchia V., Tita R. (2020). AAV-mediated FOXG1 gene editing in human Rett primary cells. Eur. J. Hum. Genet..

[113] Garg S.K., Lioy D.T., Mcgann J.C., Bissonnette J.M., Murtha M.J., Foust K.D., Kaspar B.K., Bird A., Mandel G. (2013). Systemic Delivery of MeCP2 Rescues Behavioral and Cellular Deficits in Female Mouse Models of Rett Syndrome. Neurobiol. Dis. Syst..

[114] Gadalla K.K.E., Vudhironarit T., Hector R.D., Sinnett S., Bahey N.G., Bailey M.E.S., Gray S.J., Cobb S.R. (2017). Development of a Novel AAV Gene Therapy Cassette with Improved Safety Features and Efficacy in a Mouse Model of Rett Syndrome. Mol. Methods Clin. Dev..

[115] Sinnett S.E., Hector R.D., Gadalla K.K.E., Heindel C., Chen D., Zaric V., Bailey M.E.S., Cobb S.R., Gray S.J. (2017). Improved MECP2 Gene Therapy Extends the Survival of MeCP2-Null Mice without Apparent Toxicity after Intracisternal Delivery. Mol. Methods Clin. Dev..

[116] Klein M.E., Lioy D.T., Ma L., Impey S., Mandel G., Goodman R.H. (2007). Homeostatic regulation of MeCP2 expression by a CREB-induced microRNA. Nat. Neurosci..

[117] Ribeiro A.O., Campos V.F., Lemke N., Pinhal D. (2019). Understanding the Modus Operandi of MicroRNA Regulatory Clusters. Cells.

[118] Miller D.J., Bhaduri A., Sestan N., Kriegstein A. (2019). Shared and derived features of cellular diversity in the human cerebral cortex. Curr. Opin. Neurobiol..

[119] Hodge R., Bakken T., Miller J., Smith K., Barkan E., Graybuck L., Close J., Long B., Johansen N., Penn O. (2019). Conserved cell types with divergent features in human versus mouse cortex. Nature.

[120] Anand R., Graham S.M., Hartman R.D., Forrest E.C. (2017). Sarizotan for the treatment of severe apnea in patients with rett syndrome (RTT): Rationale and design of international 6-month, randomized, placebo-controlled phase III trial (STARs). J. Neurol. Sci..

[121] Abdala A.P., Lioy D.T., Garg S.K., Knopp S.J., Paton J.F.R., Bissonnette J.M. (2014). Effect of Sarizotan, a 5-HT 1a and D2-Like Receptor Agonist, on Respiration in Three Mouse Models of Rett Syndrome. Am. J. Respir. Cell Mol. Biol..

[122] Luo C., Lancaster M.A., Castanon R., Nery J.R., Knoblich J.A., Ecker J.R., Luo C., Lancaster M.A., Castanon R., Nery J.R. (2016). Cerebral Organoids Recapitulate Epigenomic Signatures of the Human Fetal Brain Resource Cerebral Organoids Recapitulate Epigenomic Signatures of the Human Fetal Brain. Cell Rep..

[123] Chiaradia I., Lancaster M.A. (2020). Brain organoids for the study of human neurobiology at the interface of in vitro and in vivo. Nat. Neurosci..

[124] Amin N.D., Pas S.P. (2018). Building Models of Brain Disorders with Three-Dimensional Organoids. Neuron.

[125] Fedorchak N.J., Iyer N., Ashton R.S. (2020). Bioengineering tissue morphogenesis and function in human neural organoids. Semin. Cell Dev. Biol..

[126] Qian X., Song H., Ming G. (2019). Brain organoids: Advances, applications and challenges. Co. Biol..

[127] Lancaster M.A., Corsini N.S., Wolfinger S., Gustafson E.H., Burkard T.R., Otani T., Livesey F.J., Juergen A. (2017). Guided self-organization and cortical plate formation in human brain organoids. Nat. Biotechnol..

[128] Xiang Y., Tanaka Y., Cakir B., Patterson B., Kim K., Sun P., Kang Y., Zhong M., Liu X., Patra P. (2019). hESC-derived thalamic organoids form reciprocal projections when fused with cortical organoids. Cell Stem Cell..

[129] Xiang Y., Tanaka Y., Patterson B., Lee S., Weissman S.M., Park I., Xiang Y., Tanaka Y., Patterson B., Kang Y. (2017). Fusion of Regionally Specified hPSC-Derived Organoids Models Human Brain Development and Interneuron Migration Resource Fusion of Regionally Specified hPSC-Derived Organoids Models Human Brain Development. Stem Cell.

[130] Muguruma K., Nishiyama A., Kawakami H., Hashimoto K., Sasai Y. (2015). Self-Organization of Polarized Cerebellar Tissue in 3D Culture of Human Pluripotent Stem Cells. Cell Rep..

[131] Silva T.P., Bekman E., Fernandes T.G., Vaz S.H., Carlos A., Diogo M.M., Cabral J.M., Carmo-fonseca M. (2020). Maturation of human pluripotent stem cell-derived cerebellar neurons in the absence of co-culture. Front. Bioeng. Biotechnol..

[132] Nayler S., Agarwal D., Curion F., Bowden R., Becker E.B.E. (2020). Single-cell sequencing of human iPSC-derived cerebellar organoids shows recapitulation of cerebellar development. bioRxiv.

[133] Louise S., Modamio J., Mendes-pinheiro B., Sophia A., Betsou F., Christian J. (2020). Reproducible generation of human midbrain organoids for in vitro modeling of Parkinson ’ s disease. Stem Cell Res..

[134] Smits L.M., Magni S., Kinugawa K., Grzyb K., Luginbühl J., Sabate-soler S., Bolognin S., Shin J.W., Mori E., Skupin A. (2020). Single-cell transcriptomics reveals multiple neuronal cell types in human midbrain-specific organoids. Cell Tissue Res..

[135] Ogura T., Sakaguchi H., Miyamoto S., Takahashi J. (2018). Three-dimensional induction of dorsal, intermediate and ventral spinal cord tissues from human pluripotent stem cells. Co. Biol..

[136] Birey F., Andersen J., Makinson C.D., Islam S., Wei W., Huber N., Fan H.C., Metzler K.R.C., Panagiotakos G., Thom N. (2017). Assembly of functionally integrated human forebrain spheroids. Nature.

[137] Qian X., Jacob F., Song M.M., Nguyen H.N., Song H., Ming G. (2018). Generation of human brain region—Specific organoids using a miniaturized spinning bioreactor. Nature.

[138] Silva T.P., Fernandes T.G., Nogueira D.E.S., Rodrigues C.A.V., Bekman E.P., Jung S., Lee B., Carmo-fonseca M., Cabral J.M.S. (2020). Scalable Generation of Mature Cerebellar Organoids from Human Pluripotent Stem Cells and Characterization by Immunostaining. JOVE.

[139] Shi Y., Sun L., Id M.W., Id J.L., Id S.Z., Li R., Li P., Guo L., Fang A., Id R.C. (2020). Vascularized human cortical organoids (vOrganoids) model cortical development in vivo. PLoS Biol..

[140] Salmon I., Grebenyuk S., Rahman A., Fattah A., Rustandi G., Verfaillie C., Ranga A. (2021). Engineering neurovascular organoids with 3D printed microfluidic chips. bioRxiv.

